# Low-Molecular-Weight Lignin Recovery with Nanofiltration in the Kraft Pulping Process

**DOI:** 10.3390/membranes12030310

**Published:** 2022-03-09

**Authors:** Mariona Battestini Vives, Johan Thuvander, Anders Arkell, Frank Lipnizki

**Affiliations:** 1 Department of Chemical Engineering, Lund University, P.O. Box 124, 221 00 Lund, Sweden; johan.thuvander@chemeng.lth.se (J.T.); frank.lipnizki@chemeng.lth.se (F.L.); 2 RISE Innventia AB, Scheelevägen 27, 223 63 Lund, Sweden; anders@arkell.se

**Keywords:** kraft black liquor, kraft pulping process, low-molecular-weight lignin, nanofiltration

## Abstract

Kraft lignin is an underutilized resource from the pulp and paper industry with the potential of being a key raw material for renewable fuels and chemicals. The separation of high-molecular-weight lignin from black liquor by ultrafiltration has been widely investigated, while the permeate containing low-molecular-weight lignin has received little attention. Nanofiltration can concentrate the low-molecular-weight lignin. This work, therefore, evaluates nanofiltration for the separation and concentration of low-molecular-weight lignin from the ultrafiltration permeate. For this study, eight flat polymeric sheet membranes and one polymeric hollow fiber membrane, with molecular weight cut-offs ranging from 100 to 2000 Da, were tested. A parametric study was conducted at 50 °C, 2.5–35 bar, and crossflow velocity of 0.3–0.5 m/s. At a transmembrane pressure of 35 bar, the best performing membranes were NF090801, with 90% lignin retention and 37 L/m^2^·h, and SelRO MPF-36, with 84% lignin retention and 72 L/m^2^·h. The other membranes showed either very high lignin retention with a very low flux or a high flux with retention lower than 80%. Concentration studies were performed with the two selected membranes at conditions (A) 50 °C and 35 bar and (B) 70 °C and 15 bar. The NF090801 membrane had the highest flux and lignin retention during the concentration studies. Overall, it was shown that the nanofiltration process is able to produce a concentrated lignin fraction, which can be either used to produce valuable chemicals or used to make lignin oil.

## 1. Introduction

Lignin shows great promise as an upcoming raw material for renewable fuels and chemicals, such as dispersants, binders, and emulsifiers [1,2,3]. Lignin is available through kraft black liquor (KBL), a process stream within the kraft pulping process. In the mills, the KBL is concentrated in multi-effect evaporators, and then combusted in the recovery boiler, where steam is generated, and the cooking chemicals are recovered [4]. Research has shown that it is possible to recover a fraction of the kraft lignin without compromising the mill operations [5,6]. However, the lignin needs to be separated from the KBL using a suitable method before it can be used for other applications.

The main methods to separate and purify the lignin from the KBL are precipitation and membrane processes [7]. Precipitation by acidification is the most common method of extracting lignin from KBL. Usually, carbon dioxide, sulfuric acid, or waste acid from the bleaching step in the mill is used to acidify the black liquor [8,9,10]. In 1942, lignin precipitation was first commercialized by Westvaco Company, now called Ingevity [11]. In 2002, the LignoBoost process was developed in order to separate lignin from KBL in a two-stage process instead of one, which avoids the partial plugging of the filter cake. The resulting lignin contains fewer impurities than the one obtained by traditional precipitation methods [12,13]. Membrane processes have gained attention in the last decades as a separation method for lignin. Membranes allow for lignin separation without pH or temperature adjustment [14], and it is possible to control the molecular weight of the lignin fraction by using membranes with a different molecular size cut-off [15]. Nevertheless, membrane processes have not yet been commercialized for the recovery of lignin, and further research is still needed to scale up the process.

Until now, research has mainly focused on recovery of high-molecular-weight lignin by ultrafiltration (UF), while the permeate containing low-molecular-weight lignin is sent to the recovery cycle [16]. Wallberg et al. [17] investigated the possibility of using ceramic UF membranes to fractionate and concentrate lignin from KBL at 60, 75, and 90 °C. In the same research group, the performance of a ceramic membrane during concentration of softwood KBL of two Swedish pulp mills—one using batch and the other continuous digestion—was investigated [18]. UF has also been compared to microfiltration (MF) on its capability for lignin retention, finding that MF membranes had an 80% lignin retention, whereas UF membranes achieved 90% lignin retention [19]. Keyoumu et al. [20] investigated continuous separation of low-molecular-weight lignin from softwood and hardwood black liquor. Ceramic membranes in the range of nanofiltration (NF) and UF were used, and it was concluded that both are feasible methods to remove organic material from the pulp mill, and thus reduce the load to the recovery boiler. Research has also focused on the possibility of combining membrane filtration steps. Arkell et al. [21] investigated the separation of lignin and hemicelluloses from KBL using NF with or without a prior UF step. The UF step was performed with a ceramic membrane, while for the NF step, both ceramic and polymeric membranes were investigated. The preliminary economic estimation concluded that the lignin could be obtained at a lower cost using a polymeric NF membrane, without prior UF. Similarly, Dafinov et al. [22] used 5- and 15-kDa ceramic membranes to filtrate black liquor from soda-anthraquinone pulping. The collected permeate was further filtrated with a 1-kDa ceramic membrane at 5 bar. Their analysis revealed that the large macromolecules had been mostly removed in the first membrane step. Recently, SunCarbon AB, a producer of lignin-based fuels, has been working to implement a membrane filtration step consisting of UF and NF after the digester to extract lignin at full scale. The lignin extracted from black liquor is then purified and converted into a lignin-rich oil by a hydrothermal process step [23]. Additionally, researchers have also focused on NF membrane development for KBL filtration—for example, Valderrama et al. developed pH-stable NF membranes and used a KBL model solution to test their performance [24]. One of the developed membranes had a promising permeate flux of 24 L/m^2^·h at 21.5 bar, and a total organic content rejection of 92%. Wang et al. went in another direction and explored the possibility of enhancing polyether sulphone-supported membranes with graphene oxide [25]. With their membrane modification, they obtained a 99% rate of lignin rejection. Existing literature and research have thoroughly investigated UF, and considered membrane development for black liquor filtration. However, data is lacking on how commercial NF membranes would perform on recovering low-molecular-weight lignin from KBL.

In this paper, the possibility of separating low-molecular-weight lignin from KBL UF permeate by NF has been investigated. NF has great potential to separate low-molecular-weight lignin from black liquor. Moreover, low-molecular-weight lignin has the possibility of being converted into lignin-rich oil, as demonstrated by SunCarbon, which in turn can be used to produce renewable transportation fuels. The present work has evaluated nine NF membranes in parametric and concentration studies, which have been performed at laboratory scale. The membranes were evaluated based on their lignin retention and flux.

## 2. Materials and Methods

### 2.1. Feed Solution

The feed solution used for the membrane filtration tests was KBL UF permeate. The UF step was performed by SunCarbon AB (Piteå, Sweden) preceding this study; 1-kDa ceramic membranes manufactured by Alsys Kleansep (Salindres, France) were used, and the filtration took place in an on-site membrane pilot plant. The feed was a mix of softwood and hardwood KBL from a pulp and paper mill in the north of Sweden.

The KBL UF permeate had a total lignin concentration of 29 g/L, a total dry solids (TDS) content of 199 g/L, and ash content of 73 g/L. The composition and properties of the feed solution are given in Table 1. 

### 2.2. Membranes

The performance of eight polymeric flat-sheet membranes and one polymeric hollow-fiber membrane was investigated. The membranes had a molecular weight cut-off (MWCO) range from 100 to 2000 Da—thus, in or close to the NF range. The characteristics of the membranes are given in Table 2.

### 2.3. Experimental Setup

**Figure 1:**
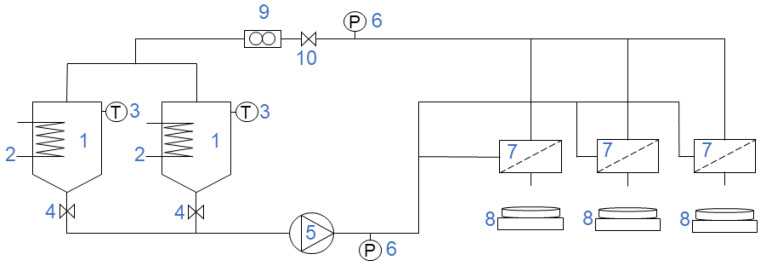
Experimental setup consisting of (1) tanks, (2) electric heaters, (3) temperature sensors, (4) valves, (5) feed pump, (6) manometers, (7) membrane modules for flat-sheet membranes, (8) digital scales, (9) flowmeter, and (10) retentate valve.

The experimental setup (Figure 1) used in this study consists of three flat-sheet membrane modules connected in parallel or a hollow-fiber module. In total, two different tanks were used: a 15-L tank for the feed, and a 5-L tank for the cleaning solution and the sodium hydroxide solution. The solutions were fed to the system by a pump (Hydra-cell D25XL, Wanner, Minneapolis, MN, USA), which was connected to a frequency converter (ELEX 4000, Bergkvist and Co. AB, Gothenburg, Sweden) controlling the flow rate. A temperature regulator (MCM-100, Shinko Technos Co., Ltd., Osaka, Japan) was controlling the temperature in the tanks using a sensor (Pt 100, Pentronic, Västervik, Sweden) and electrical heaters, both immersed in the tanks. The permeate mass was measured with electronic scales (PL6001-S, Mettler-Toledo AB, Stockholm, Sweden). The flow was monitored by a flowmeter (FCH-34-PP-Chemica, B.I.O-TECH e.K., Vilshofen an der Donau, Germany). The transmembrane pressure (TMP) was regulated by a valve placed on the retentate side of the modules. The pressure was measured by two manometers (DCS 8864, Trafag, Bubikon, Switzerland): one at the feed side, *P_before_*, and one at the retentate side, *P_after_*. The TMP is given by Equation (1):

(1) $$\mathrm{TMP}=\frac{P_{before}+P_{after}}{2}-P_{permeate}$$

The temperature, pressure, permeate flux, flow, and crossflow velocity (CFV) were monitored and recorded using LabVIEW 2017 (National Instruments, Austin, TX, USA).

For the parametric studies with flat-sheet membranes, the experimental setup shown in Figure 1 was used. A circular flat-sheet membrane with an area of 0.00196 m^2^ was placed in each module. The membranes were tested in groups of three according to their MWCO. The same setup was used for the concentration studies, with the change that only two of the flat-sheet membrane modules were used, and thus, two different membranes were tested at the same time.

In the experiments with the hollow-fiber membrane, the flat-sheet modules were replaced with a single hollow-fiber module. The membrane area was 0.067 m^2^. 

### 2.4. Experimental Procedure

#### 2.4.1. Cleaning and PWF Measurements

All membranes were cleaned for 1 h before and after each experiment using a 0.1 wt% solution of the cleaning agent Ultrasil 110 (Ecolab AB, Älvsjö, Sweden). The temperature for the cleaning was 50 °C, the pressure was 2 bar, and the CFV was 0.5 m/s. After the cleaning, the membranes were rinsed thoroughly with deionized water to remove the cleaning agent from the system. The pure water flux (PWF) was measured at 50 °C and at three different TMPs: 5, 10, and 15 bar. The PWF was measured after the first cleaning, after the concentration study, and after the second cleaning. 

The membrane permeability (L/m^2^·h·bar) was calculated by dividing the PWF by the applied TMP, and the average was calculated for the three TMPs. The membrane permeability after each concentration study and after the second cleaning was normalized to the permeability after the first cleaning of the new membrane sample. 

#### 2.4.2. Sodium Hydroxide Conditioning

Sodium hydroxide (NaOH) conditioning was performed before and after KBL filtration to avoid a sudden pH change. The feed tank was filled with 5 L of 0.1 M NaOH solution (Merck KGaA, Darmstadt, Germany), and the solution was recirculated within the system while the temperature was slowly increased to 50 °C. Once the temperature was reached, KBL was fed into the system to start the filtration. At the end of the parametric and concentration studies, the system was flushed with the same sodium hydroxide solution before changing the feed to deionized water.

#### 2.4.3. Parametric Studies

For the parametric studies, the retentate and permeate were recirculated back to the feed tank in order to keep the concentration constant. The temperature was 50 °C, and the CFV was 0.5 m/s. The TMP was increased to 5, 10, 15, 20, 25, 30, and 35 bar, measuring the flux for approximately 30 min before withdrawing a permeate sample at each TMP. Samples were taken from the feed tank at the start and at the end of each KBL filtration. New membranes were used for each parametric study performed. 

The observed lignin retention was calculated for each membrane at the defined TMPs according to Equation (2):

(2) $$R_{obs}\left(\%\right)=\left(1-\frac{C_{p}}{C_{f}}\right)\cdot 100$$

where *C_p_* is the lignin concentration in the permeate and *C_f_* is the lignin concentration in the feed. 

#### 2.4.4. Concentration Studies

The membranes found to be most suitable in the parametric studies were used in a concentration study. For this study, two sets of conditions were used: (A) 50 °C and 35 bar and (B) 70 °C and 15 bar. The CFV was 0.5 m/s. Condition A was chosen because the membranes found to be the most suitable had both the highest flux and lignin retention at this specific pressure and temperature. Condition B was chosen because the KBL stream in the mill is usually at a higher temperature than 50 °C, and hence, energy for cooling can be saved if the membranes can operate at higher temperature. New membranes were used for each concentration study performed.

The initial feed volume in the tank was 12 L of KBL UF permeate. Both retentate and permeate were initially recirculated to the feed tank, while the TMP was gradually increased to the final operating pressure. During the recirculation, the feed solution was slowly heated. Once pressure, temperature, and CFV were stable, the concentration in the feed tank was increased by constant withdrawal of the permeate while recirculating the retentate. The concentration studies were stopped once the permeate flux was 5 L/m^2^·h or lower for at least one of the membranes. Initial permeate samples were taken 1 h after starting the concentration study, and the other permeate samples were taken at regular intervals. Retentate samples were taken from the feed tank at the same time as the permeate samples. The volume reduction (VR) was calculated for each set of samples taken and is defined as the ratio between the volume of the permeate (*V_p_*) and the initial feed volume (*V*_0_), and is expressed as a percentage (Equation (3)):

(3) $$\mathrm{VR}\;\left(\%\right)=\frac{V_{p}}{V_{0}}\cdot 100$$

#### 2.4.5. Analysis

The analysis performed was adapted from the standardized NREL Laboratory Analytical Procedures [26,27]. The TDS were determined by drying samples in previously weighed crucibles at 105 °C for 24 h. After drying, the samples were cooled down in a desiccator for 30 min and weighed. The ash content was determined by first heating to 575 °C, and then at 900 °C. The samples were placed in a muffle furnace (Nabertherm, Lilienthal, Germany), and then the temperature was heated to 575 °C and maintained for 3 h. After, the samples were heated to 900 °C and maintained for 3 h. Then, the crucibles were cooled down to room temperature in a desiccator for 30 min and weighed. The ash content was calculated from the weight of the residue. TDS and ash content analysis was carried out three times per feed sample (Table 1). 

The total lignin content was determined by measuring the UV absorbance at a 280-nm wavelength in a spectrophotometer (UV-160, Shimadzu Corp., Kyoto, Japan). Samples were diluted using 0.1 M NaOH solution to reach an absorbance between 0.65 and 0.85. A weighted absorption coefficient of 23.6 g/L·cm was used. This was the result of weighting the hardwood adsorption coefficient of 21.1 g/L·cm [28], and the softwood absorption coefficient of 24.6 g/L·cm [21] with the percentages of hardwood and softwood used at the pulp mill. 

To characterize the feed, acid hydrolysis was performed to determine the content of acid-insoluble lignin and sugars according to ‘determination of structural carbohydrates and lignin in biomass’ [26]. The acid hydrolysis degraded the hemicelluloses to monomeric sugars, and their quantity was determined using high-performance anion exchange chromatography coupled with pulsed amperometric detection (ICS-3000, Dionex Corporation, Sunnyvale, CA, USA), which was equipped with a Carbo Pac PA1 analytical column (Thermo Fisher Scientific, Waltham, MA, USA). The injection volume was 10 µL. Deionized water was used as eluent at a flow rate of 1 mL/min, and 170 mM sodium acetate in 200 mM sodium hydroxide was used as a cleaning solution. The standards used for calibration were L-arabinose (VWR International, Radnor, PA, USA), D-galactose (Merck KgaA, Darmstadt, Germany), D-glucose (VWR International), D-xylose (ITW Reagents, Castellar del Vallès, Spain), and D-mannose (Alfa Aesar, Haverhill, MA, USA).

The pH of the KBL UF permeate was analyzed with a Hanna HI 8424 pH meter (Hanna Instruments, Woonsocket, RI, USA), which had an HI-11310 electrode from the same manufacturer.

## 3. Results and Discussion

The Section 3 is divided into two main parts. In the first part, the parametric studies’ results are presented and discussed. In the second part, the results and implications of the concentration studies performed with the membranes selected during the parametric studies will be revealed.

### 3.1. Parametric Studies

#### 3.1.1. Influence of TMP on the Flux

**Figure 2:**
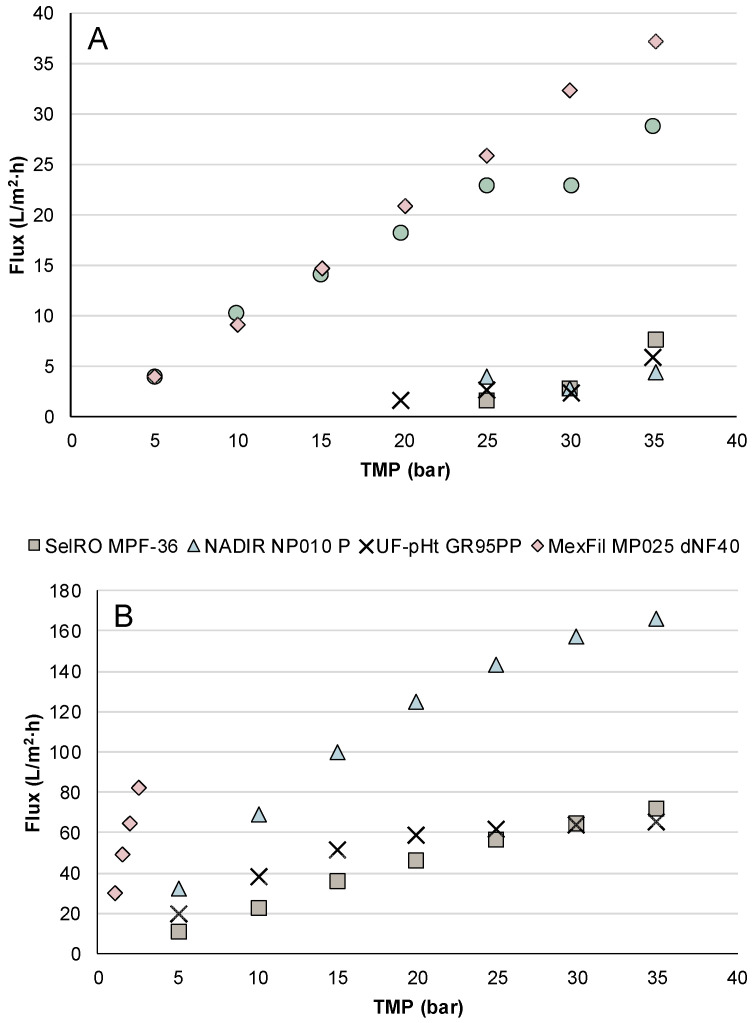
Permeate fluxes of the nine membranes tested during the parametric studies at different TMPs for (**A**) membranes NanoPro B-4021, NanoPro B-4022, NF090801, NADIR NP030 P, and SelRO MPF-34, and (**B**) membranes SelRO MPF-36, NADIR NP010 P, UF-pHt GR95PP, and MexFil MP025 dNF40.

The influence of TMP on the flux for the nine membranes is shown in Figure 2. For the polymeric membranes NanoPro B-4021 and NanoPro B-4022, no permeate was produced until a TMP of 25 bar was reached. Similarly, the SelRO MPF-34 membrane only started to produce permeate at a TMP of 20 bar. At a TMP of 35 bar, which was the highest TMP applied, the AMS polymeric membranes NanoPro B-4021 and NanoPro B-4022 had a flux of 7 L/m^2^·h and 4 L/m^2^·h, respectively. This was likely due to the small MWCO of the NanoPro B-4021, NanoPro B-4022, and SelRO MPF-34 membranes, which was in the range of 100–200 Da (Table 2). Due to their small MWCO, a higher driving force is needed for these denser membranes to allow solutions to pass through. The NADIR NP030 P and the NF090801 have slightly higher MWCOs, 500–600 Da and 350 Da, respectively, but have a much higher flux in comparison.

The highest flux, 165 L/m^2^·h, was obtained with the NADIR NP010 P membrane at 35 bar. The other polymeric membranes had lower permeate fluxes than that of the NP010, which was expected, as the NP010 had one of the largest MWCOs of all the polymeric flat-sheet membranes tested. UF-pHt GR95PP and SelRO MPF-36 had similar MWCOs as the NADIR NP010 P, but their flux was much lower (Figure 2B). For the UF-pHt GR95PP, the flux stopped increasing around TMP 20 bar, whereas for the SelRO MPF-36, the flux continued to increase when TMP increases, but at a slower rate compared to the NP010. One explanation for this phenomenon could be that an increase in TMP, and thus, in driving force, caused the convective transport of solute molecules towards the membrane surface to be greater. Therefore, the amount of solute molecules reaching the membrane surface was higher and concentration polarization, and overtime, fouling increased, which caused a decrease in the permeate flux.

The MexFil hollow-fiber membrane had a permeate flux of 82 L/m^2^·h at 2.5 bar, the highest set TMP for this membrane. The TMP was not increased further due to limitations of the membrane. Even with pressure limitations, the polymeric hollow-fiber membrane had a higher flux than the polymeric flat-sheet membranes with similar MWCOs. The differences may be due to the difference in membrane geometry. 

#### 3.1.2. Lignin Retention

**Figure 3:**
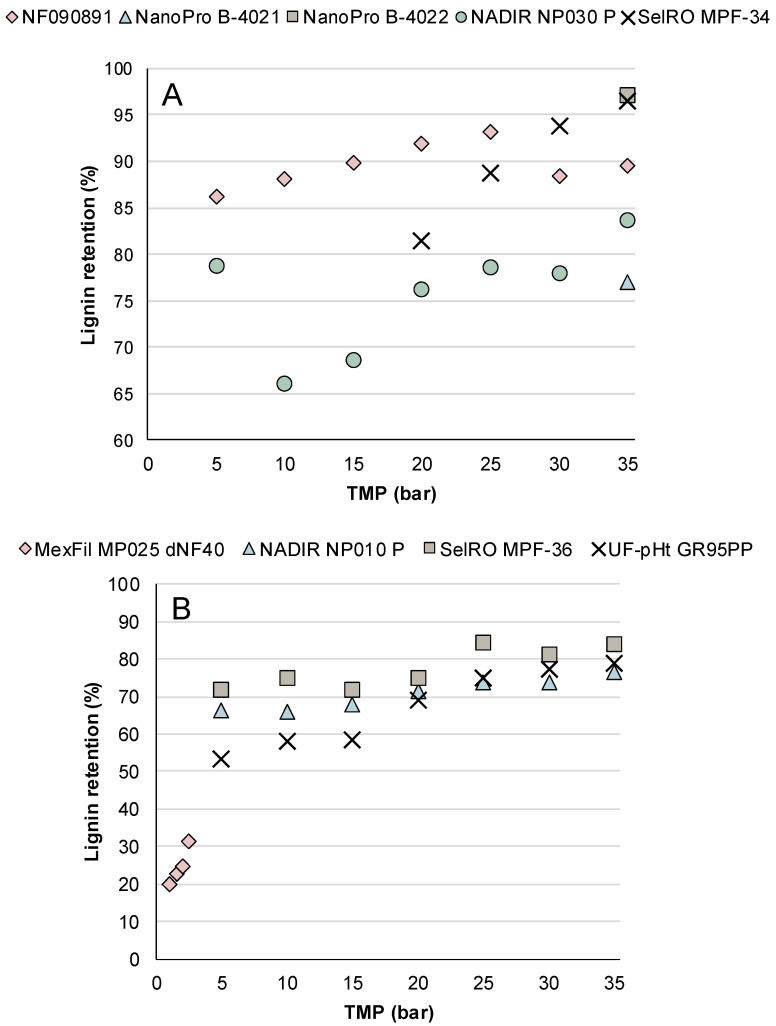
Lignin retention in percentage at different TMPs for (**A**) membranes NanoPro B-4021, NanoPro B-4022, NF090801, NADIR NP030 P, and SelRO MPF-34, and (**B**) membranes SelRO MPF-36, NADIR NP010 P, UF-pHt GR95PP, and MexFil MP025 dNF40.

The total lignin retention for the membranes at different TMPs is shown in Figure 3. All membranes tested at TMPs higher than 25 bar showed a lignin retention higher than 70%. As seen in Section 3.1.2, a high TMP usually causes a high flux. However, high TMPs might cause the membranes to compact, reducing the pore size, and hence increasing lignin retention. Long exposure of increasing TMP might also result in the formation of a cake layer on the membrane surface, which in turn leads to a higher retention of lignin. 

The highest lignin retention obtained was 97%, achieved by the NanoPro B-4022 membrane, closely followed by the SelRO MPF-34 membrane, with 96% lignin retention, both at a TMP of 35 bar (Figure 3A). Since these two are denser membranes, lignin retentions close to 100% were expected. NanoPro B-4021, despite having a similar MWCO and flux compared to NanoPro B-4022 and SelRO MPF-34, had a lower lignin retention of 77%. NF090801 showed a high lignin retention of 86% at 5 bar, and the retention increased up to 96% when the TMP was increased to 25 bar. However, the same membrane had a slightly lower lignin retention, 88% and 90% at 30 and 35 bar, respectively. NADIR NP030 P had a similar flux behavior as the NF090801 but a lower lignin retention, specifically between TMPs of 10 and 20 bar, when it ranged from 66 to 76%. NP030 had a slightly higher MWCO than NF090801, which could explain why the lignin retention was lower. 

The MexFil hollow-fiber membrane exhibited the lowest lignin retention, 31% at 2.5 bar, likely in part due to the low TMP used compared to the other membranes (Figure 3B). NADIR NP010 P, with one of the biggest MWCOs of the tested membranes, had the lowest lignin retention at 35 bar: 76%. The other two membranes in a similar MWCO range were the SelRO MPF-36 and the UF-pHt GR95PP, which had a similar lignin retention, even though they had lower flux. SelRO MPF-36 membrane reached a lignin retention higher than 80% when the TMP was above 25 bar, while the UF-pHt GR95PP had a lignin retention higher than 75% when the TMP was above 25 bar. Membrane compaction could explain why these two membranes have a high lignin retention but a lower flux than expected for their MWCO. 

As showed by these results, MWCO is not an absolute parameter that determines the retention in NF. Other aspects, such as adsorption, concentration polarization, pressure dependency, and feed concentration, might have affected the retention of the lignin.

Both high lignin retention and high flux are important when evaluating membranes for lignin recovery from KBL. Therefore, membranes with high flux and a lignin retention over 80% were considered for the concentration study. The membranes that showed these characteristics were SelRO MPF-36 and NF090801. Compared to the other membranes that have a high lignin retention, NF090801 and SelRO MPF-36 also had relative high fluxes of 37 L/m^2^·h and 72 L/m^2^·h, respectively, at a TMP of 35 bar. The fluxes obtained for these two membranes are in line with the literature [21,29]. Thus, these two membranes were selected for concentration studies, the results of which are presented in the next section. 

### 3.2. Concentration Studies

**Figure 4:**
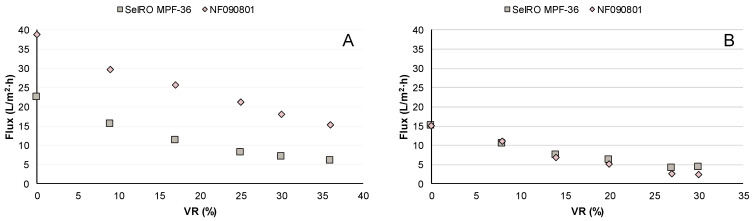
Flux vs VR during the concentration studies with the SelRO MPF-36 and NF090801 membranes at (**A**) condition A, 50 °C and 35 bar, and (**B**) condition B, 70 °C and 15 bar.

A total of two concentration studies were performed with both SelRO MPF-36 and NF090801. The conditions for the studies were (A) 50 °C and 35 bar and (B) 70 °C and 15 bar, both with a CFV of 0.5 m/s. The first study took 51 h to reach VR 35%, while the second study took 54 h to reach VR 30%. The flux-as-a-function-of-VR results can be seen in Figure 4.

The initial permeate flux of the NF090801 membrane was 38 L/m^2^·h using condition A, which was more than double than the flux at condition B, 15 L/m^2^·h. The difference in starting flux might be due to the TMP applied. As discussed in Section 3.1., flux increases with increasing TMP. The flux at condition A for NF090801 was in line with the one obtained during the parametric studies, which was 37 L/m^2^·h. 

The starting permeate fluxes of the SelRO MPF-36 membrane for both concentration studies were quite similar: 23 L/m^2^·h for condition A and 15 L/m^2^·h for condition B. The final fluxes were approximately 5 L/m^2^·h in both studies. It appears that SelRO MPF-36 performance remained similar when operated at different temperature and pressure conditions. It was therefore difficult to assess which of the two parameters affected the membrane performance the most. In this case, the starting flux at condition A for SelRO MPF-36 was smaller than the one from the parametric studies, which was 72 L/m^2^·h. The difference in flux could be explained by membrane compaction. Before the concentration study, the system was stabilized for 1 h before starting to withdraw permeate. 

A flux decrease at similar rates was observed during both concentration studies. During a concentration study, there was an increase in bulk concentration which consequently caused a decrease in the mass transfer coefficient, and in turn, there was a decrease in flux. At the end of the second study, the fluxes were lower than 5 L/m^2^·h for both membranes, and the volume reduction did not increase. Because, at 70 °C and 15 bar, the starting fluxes for both membranes were lower, lower fluxes were reached much earlier than at 50 °C and 35 bar, specifically for the SelRO MPF-36.

#### 3.2.1. Lignin Retention

**Figure 5:**
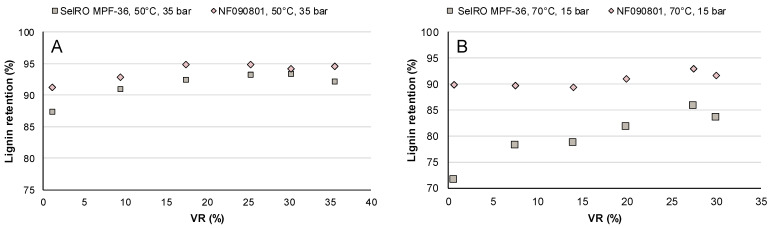
Lignin retention vs VR during the concentration studies with the SelRO MPF-36 and NF090801 membranes at (**A**) condition A, 50 °C and 35 bar, and (**B**) condition B, 70 °C and 15 bar.

Lignin retention was plotted against volume reduction, and the results can be seen in Figure 5. 

The first concentration study, performed at 50 °C and 35 bar, delivered the best lignin retentions for both membranes. The lignin retention for the NF090801 varied between 91 and 94%, whereas the retention for the SelRO MPF-36 membrane initially increased from 87 to 93% at VR 25%, and then, it was stable for the remaining time of the study. The lignin retention of the NF090801 and the SelRO MPF-36 was slightly higher than the one obtained during the parametric studies at TMP 35 bar. Constant high TMP might have facilitated the formation of a cake layer and increased the lignin retention, even if the operating conditions were the same. 

In the second concentration study, performed at 70 °C and 15 bar, the NF090801 delivered slightly lower lignin retention, between 89 and 92%, compared to the first study. On the other hand, the lignin retention for SelRO MPF-36 started at 70% and increased to 85%. For this membrane, the lignin retention was not as good as it was at a higher TMP.

In the studied cases, a higher TMP seemed to favor a higher lignin retention, and the temperature seemed to have a minor effect. In this case, in condition B, the temperature was increased in comparison to condition A, but then the TMP was lowered. The increase in temperature did not compensate for the decrease in pressure. The NF090801 presented the best lignin retention in both conditions, although at 50 °C and 35 bar, it had the highest lignin retention and flux.

#### 3.2.2. Membrane Permeability and Fouling

**Figure 6:**
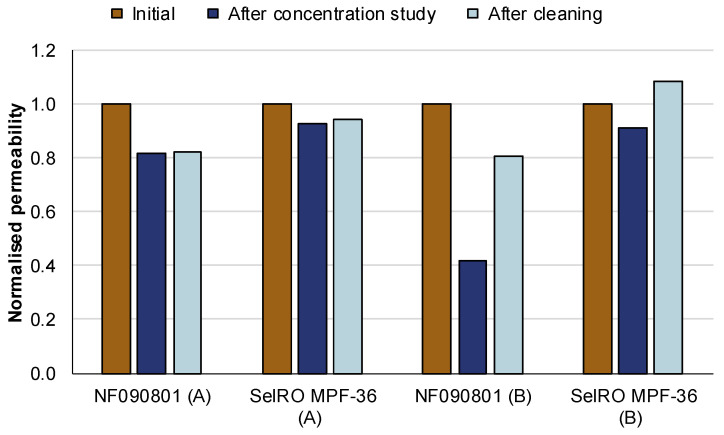
Normalized permeability before the concentration study (initial), after the concentration study and flushing with NaOH (after concentration study), and after cleaning with Ultrasil 110 (after cleaning) at condition A, 50 °C and 35 bar, and condition B, 70 °C and 15 bar.

The fouling of the membranes during the concentration studies at different conditions was evaluated with the normalized permeability of the PWF measurements. Figure 6 shows the normalized permeability for both membranes used during the concentration studies initially (PWF with pristine membrane), after the concentration study, and after being cleaned with Ultrasil 110.

The cleaning protocol used during the concentration studies seems to have been successful in recovering the flux of both membranes after the concentration studies. For condition A, the permeability after the concentration study and after cleaning was 80–90% of the initial permeability for both membranes. For condition B, the permeability of the membrane NF090801 after the concentration study was 40% of the initial permeability, which could be recovered to 80% after cleaning. In comparison, the SelRO MPF-36 had minor fouling after the concentration study, as its permeability was reduced only 10%. However, after cleaning, the permeability of the SelRO MPF-36 increased to 110%, which theoretically should not be possible. This indicates that the first cleaning performed did not properly remove all the storage chemicals from the membrane. However, after the concentration study, and flushing with NaOH solution and water several times, all the chemicals were successfully removed, and the actual permeability of the membrane was obtained. Another possible explanation is that the high pH and temperature during the filtration damaged the membrane pores, hence, increasing their size. Nevertheless, signs of this would have been seen by an increase of the flux during the concentration study, which was not the case. Therefore, the most likely explanation is the removal of storage chemicals.

The SelRO MPF-36 membrane seems to be more resistant to fouling by KBL than the NF090801 membrane. However, even with the higher resistance to fouling, the flux for the NF090801 was higher during both conditions.

## 4. Conclusions

This study demonstrated that NF membranes can favorably recover low-molecular-weight lignin if coupled with a previous UF step. A total of nine NF membranes were evaluated in regards to their flux and lignin retention, and it was found that the polymeric flat-sheet membranes had a high lignin retention, ranging from 70% to 97% at the highest TMP, 35 bar. 

The membranes with the best combination of flux and lignin retention were chosen for concentration studies: NF090801 and SelRO MPF-36. A VR of 35% was obtained when operating at 50 °C and 35 bar, which achieved a maximum lignin retention of 94% for the NF090801, and 92% for the SelRO MPF-36 membrane. In comparison, a VR of 30% was obtained when operating at 70 °C and 15 bar, which achieved a maximum lignin retention of 92% for the NF090801 and 85% for the SelRO MPF-36. The flux was higher during the low temperature and high TMP conditions, likely as the lower viscosity at the higher temperature did not compensate for the reduced TMP.

The cleaning protocol used during this study was successful in recovering at least 80% of the flux of both NF090801 and SelRO MPF-36. Therefore, alkaline cleaning seems to be effective for foulants found in KBL UF permeate.

After performing both permeate and concentration studies, it was found that the membrane with the most successful combination of flux and lignin retention was the NF090801. Further research into scaling up the NF step is needed, as well as characterization of the retentate with high concentrations of low-molecular-weight lignin. Additionally, the low-molecular-weight lignin concentrate obtained using the membrane could then be properly treated to separate the lignin and convert it to a value-added chemical.

## Figures and Tables

**Table 1:** Feed solution composition and properties. The analyses were performed in triplicate.

Data	KBL UF Permeate
pH	13.13 ± 0.08
TDS (g/L)	199.14 ± 2.44
Ash (g/L)	73.59 ± 1.38
Total hemicelluloses (g/L)	2.01
Arabinose (g/L)	0.44 ± 0.05
Galactose (g/L)	1.19 ± 0.05
Glucose (g/L)	0.11 ± 0.03
Xylose (g/L)	0.27 ± 0.05
Total lignin (g/L)	29.72 ± 0.81
Klason lignin (g/L)	22.78 ± 0.32

**Table 2:** Nanofiltration membranes tested and their characteristics. MWCO and material are shown as specified by the manufacturer in the data sheet of the membrane.

Name	Manufacturer	Type	MWCO (Da)	Material
MexFil MP025 dNF40	NXFiltration, Enschede, The Netherlands	Hollow-fiber	400	Modified PES
UF-pHt GR95PP	Alfa Laval, Nakskov, Denmark	Flat-sheet	2000	PES
NADIR NP030 P	MANN+HUMMEL Water & Fluid Solutions, Wiesbaden, Germany	Flat-sheet	500–600	PES
NADIR NP010 P	MANN+HUMMEL Water & Fluid Solutions, Wiesbaden, Germany	Flat-sheet	1000–2000	PES
SelRO MPF-36	Koch Separation Solutions, Wilmington, MA, USA	Flat-sheet	1000	Not specified
SelRO MPF-34	Koch Separation Solutions, Wilmington, MA, USA	Flat-sheet	200	Not specified
NF090801	SolSep BV, Apeldoorn, The Netherlands	Flat-sheet	350	PES
NanoPro B-4021	AMS Technologies, Or Yehuda, Israel	Flat-sheet	100	Not specified
NanoPro B-4022	AMS Technologies, Or Yehuda, Israel	Flat-sheet	150	Not specified

## Data Availability

Not applicable.
